# Regulatory provisions for post-release monitoring of genetically modified organisms in Africa

**DOI:** 10.3389/fbioe.2026.1785298

**Published:** 2026-04-10

**Authors:** Julia Njagi, John Muriuki, Paul Mbugua, Brinda Dass, Stephanie James, Dorington Ogoyi

**Affiliations:** 1 Biosafety Licensing, Monitoring and Surveillance, National Biosafety Authority, Nairobi, Kenya; 2 Department of Environmental Science and Education, Kenyatta University, Nairobi, Kenya; 3 Department of Plant Sciences, Kenyatta University, Nairobi, Kenya; 4 GeneConvene Global Collaborative, Foundation for the National Institutes of Health (FNIH), North Bethesda, MD, United States; 5 Department of Biological and Life Sciences, Technical University of Kenya, Nairobi, Kenya

**Keywords:** commercialization, environmental protection goals, GMOs, post-release monitoring, regulatory frameworks

## Abstract

Genetically modified (GM) crops with improved traits such as resistance to biotic and abiotic stresses and enhanced nutritional profiles have been commercially cultivated for over three decades. Despite extensive safety data and long-term cultivation experience, concerns continue to be raised about the potential risks and benefits of genetically modified organisms (GMOs). This is particularly true for the African region, where only eight out of fifty-four countries have so far commercialized GMOs. Upon release into the environment, GM crops may interact with ecosystems in complex ways, possibly leading to unanticipated ecological effects. Consequently, post-release monitoring of GMOs is essential to identify early signs of adverse impacts, enabling timely responses such as adjustments in risk management strategies, mitigation measures, or re-evaluation of previous regulatory decisions. A supportive policy and regulatory environment are critical for facilitating the safe development, testing, and commercialization of GMOs. This study conducted a desktop review of post-release monitoring frameworks for GMOs in selected African countries, as well as interviews with key informants in countries that have commercialized at least one GMO product. The findings reveal that most sampled countries lack clearly defined environmental protection goals and specific provisions regarding the scope and duration of post-release monitoring of GMOs. Where the duration of monitoring is prescribed, it is a blanket cover for all GMOs regardless of their life cycles. Moreover, the responsibility for monitoring is often delegated entirely to the applicant, and where local institutions are involved, there is no clear coordination mechanism for data sharing. These findings underscore the need for case-by-case monitoring approaches, guided by clearly articulated national protection goals and clear roles and coordination among stakeholders to ensure the safe and responsible deployment of GMOs.

## 1 Introduction

The number of countries that have commercially cultivated GMOs has continued to increase since the introduction of the first GM crop in 1996. In 2024, 27 countries planted a total of 10 different GM crops, with soybean leading at 105.1 million hectares, followed by maize (68.4 million hectares) and cotton (24.8 million hectares). The area under GM crops globally increased by 1.9% over the previous year to reach 209.8 million hectares. The leading countries with the highest acreage of GM crop cultivation in 2024 included the USA, Brazil, Argentina, Canada, and India, respectively (AgbioInvestor, 2025). In Africa, the following countries have commercialized GM crops: South Africa (maize, cotton, soyabean), Nigeria (cotton, cowpea, and maize), Ghana (cowpea), and Bt cotton in Sudan, Malawi, Eswatini, Kenya, and Ethiopia (Wesseler et al., 2017; ABNE, 2025).

Most African biosafety regulatory frameworks require a thorough risk assessment on food/feed safety, environmental safety, and socio-economic considerations before approving the introduction of GMOs into the environment (Adenle et al., 2013; McLean et al., 2003). A key part of this approval process for environmental release is conducting an environmental risk assessment in line with Annex III of the Cartagena Protocol on Biosafety to the Convention on Biological Diversity (CPB, SCBD, 2000). This science-based process begins with hazard identification, which is part of problem formulation. It involves identifying plausible risk scenarios and hypotheses to assess whether the specific GMO could cause adverse effects on the evaluation endpoints serving as proxies for protection goals. This is done by checking if any new traits of the GMO or its intended use could result in potential harm to the receiving environment. The subsequent steps include exposure assessment, hazard characterization, risk characterization, and deciding if the risks are acceptable or manageable, including strategies to address any risks if needed (Bauer-Panskus et al., 2020). Food safety assessment typically follows the guidelines of CODEX Alimentarius, focusing on comparing the GMO with a non-GMO counterpart for allergenicity, toxicity, and nutritional content (FAO/WHO Codex Alimentarius Commission, 2003). While Article 26 of the CPB states that countries may consider socio-economic factors, many African nations require that socio-economic considerations be included. In most African countries, the specific socioeconomic issues to be addressed are outlined in national guidance documents. In Kenya, for example, socio-economic considerations arising from the impact of the GMOs on the environment are mandatory (Kenya, 2009). Issues such as coexistence, access to the technology, trade implications, freedom of choice, and income security, among others, are considered during the decision-making process (Falck-Zepeda, 2009; Koch et al., 2025).

The Convention for Biological Diversity (CBD), in Article 7, states that “Parties to the Convention shall, as far as possible and as appropriate, monitor the components of biological diversity important for its conservation and sustainable use, and identify activities that are likely to have significant adverse impacts” (United Nations, 1992). Annex III 8 (f) of CPB on risk assessment outlines that in instances where there is uncertainty regarding the level of risk, this may be addressed by requesting further information on the specific issues of concern or by implementing appropriate risk management strategies and/or monitoring the GMOs in the receiving environment. Further guidance is provided by the CBD guidance document on risk assessment and monitoring of GMOs, which provides for case-specific monitoring (CSM) and general surveillance (GS) (CBD, 2016). CSM may be conducted to address uncertainty in the level of risk for effects anticipated in the risk assessment and is, therefore, hypothesis-driven. It is aimed at gathering further information to address uncertainties on the level of risk or to confirm that the conclusions of the risk assessment were accurate once the environmental release has taken place (CBD, 2016). In instances where risk management strategies were implemented along with an environmental release, monitoring may be used to evaluate the effectiveness of these risk management strategies. GS is used to account for effects that were not anticipated in the risk assessment. It starts with general observations of changes in indicators and parameters, such as assessment endpoints, which are often defined within national protection goals or are related to the conservation and sustainable use of biological diversity, taking into account risks to human health (CBD, 2016). Several countries have made it mandatory as a key component of their biosafety Regulations to carry out post-release monitoring of approved GMOs aimed at identifying any potential, unforeseen long-term adverse effects on human health or the environment (EFSA, 2011; Dolezel et al., 2024).

Monitoring facilitates timely response actions such as adjusting risk management strategies, implementing emergency plans, conducting new risk assessments, or re-evaluating previous decisions (CBD, 2016). This study was conducted to assess how African countries that have already domesticated the CPB and are at different stages of implementation have prepared for post-release GMO monitoring. The research included desktop reviews of publicly available regulatory policies, including those in the CBD Biosafety Clearing House (https://bch.cbd.int/en/) and the National Clearing Houses, along with relevant publications. Additionally, surveys were conducted in the targeted countries to address identified gaps.

## 2 Methodology

This study employed a systematic qualitative review methodology to assess and compare regulatory provisions governing post-release monitoring of GMOs in 26 African countries. The countries included in the study were from the Eastern and Central African region (Kenya, Uganda, Tanzania, Rwanda, Ethiopia and Cameroon), Southern African Region (South Africa, Malawi, Zambia, Eswatini, Mozambique, Botswana, Namibia and Zimbabwe), Western African region (Nigeria, Ghana, Burkina-Faso, Mali, Senegal, Benin, Ivory Coast, Togo and Niger) and Northern African region (Egypt, Sudan and Tunisia). The review undertook a comparative approach to analyze policy documents and published literature, aiming to understand how biosafety regulatory frameworks in the above countries have addressed the potential impacts of GMOs following their environmental release. This structured approach sought to identify whether post-release monitoring was explicitly included in their national biosafety regulatory frameworks, establish the scope and duration, and allow for thematic comparison across jurisdictions and contextual insights into national biosafety governance. Consequently, the study also looked at the effectiveness of such provisions, highlighted gaps, and made recommendations for best practices. Data sources included extensive desktop reviews of biosafety laws and regulations and related documents accessed through the internet and the Biosafety Clearing House. Additionally, a questionnaire was developed and shared with key informants to probe the regulatory frameworks further. In some instances, face-to-face meeting interviews with regulators, emails, and phone calls were also employed.

The inclusion criteria for countries under review were that they were Parties to the Protocol, had put in place a mechanism for its domestication by having a national biosafety regulatory framework, had commercialized GMOs, and or evidence of GMO research activities being undertaken or previously undertaken. A structured matrix was developed to extract and compare core elements of post-release monitoring requirements/frameworks across the selected countries. To assess the adequacy of post-release monitoring frameworks, country-specific provisions were benchmarked against the CPB (especially Article 16 and practices from countries with experience in post-release monitoring, such as Brazil and the European Union (EU). The results from the survey and interviews were confirmed through triangulation, which involved comparing the insights from the interviews with the survey data and examining relevant policy documents from the participating countries to ensure a thorough and reliable interpretation.

## 3 Results

**FIGURE 1:**
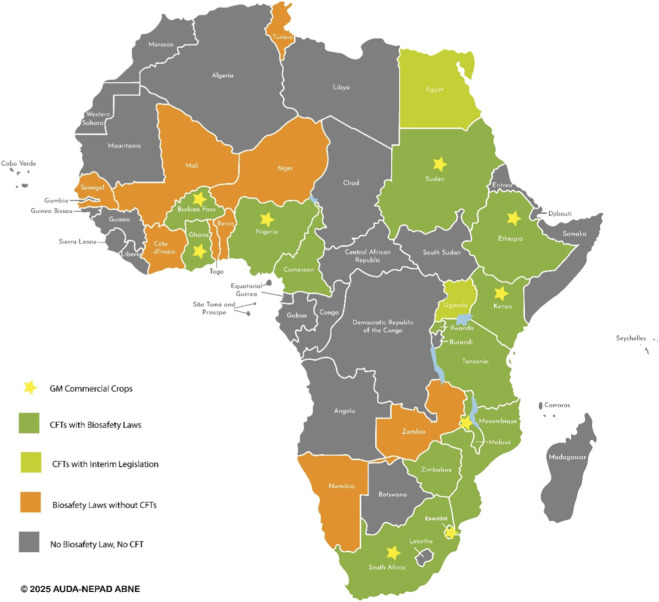
Biotechnology regulatory status in Africa (https://www.nepad.org/publication/map-of-biotechnology-regulatory-status-africa).

African countries are at varying stages of implementation and adoption of GMOs, as illustrated in the map below (Figure 1). Fifty-one (51) of the fifty-four (54) countries in Africa are Parties to the CPB, with a large number of countries having developed comprehensive national biosafety regulatory frameworks and enacted laws to guide the adoption of GM technology. Countries including Zambia, Mali, Senegal, Niger, Benin, Namibia, and Côte d'Ivoire have laws but are not utilizing the technology. GM Field trials are being carried out in Uganda, Rwanda, Tanzania, Zimbabwe, Egypt, Mozambique, and Cameroon. A handful of countries, including South Africa, Sudan, Malawi, Eswatini, Nigeria, Burkina Faso, Ethiopia, Ghana, and Kenya, have experience with the commercial cultivation of GMOs (Wesseler et al., 2017; ABNE, 2025; Sadikiel Mmbando, 2024).

### 3.1 Provisions for post-release monitoring in eastern and central Africa

In this region (see methodology), confined field trials (CFT) for several GM crops have been carried out in all countries except Cameroon. In Kenya, CFTs for GM crops addressing various agricultural constraints have been carried out for cotton, maize, sorghum, sweet potato, cassava, bananas, gypsophila flower, and potatoes (http://ke.biosafetyclearinghouse.net/approvedcft.shtml). Research with GM animals and animal health inputs is also ongoing in Kenya, where vaccines against livestock diseases like East Coast Fever, Rift Valley Fever, and *Mycoplasma mycoides* are under ongoing research under containment (http://ke.biosafetyclearinghouse.net/approvedcontaineduse.shtml). GM animals like trypanotolerant cows and goats are also under research. Insect-resistant cotton received approval for commercialization in Ethiopia and Kenya in 2019 and 2020, respectively (Komen et al., 2020; ISAAA, 2025a). In 2025, the Ethiopian National Variety Release Committee approved the commercial release of three TELA maize hybrid varieties genetically modified for improved insect resistance and drought tolerance (USDA, 2025). Additionally, Kenya authorized the environmental release of insect-resistant maize in 2022 and National Performance Trials for virus-resistant cassava in 2021 (http://ke.biosafetyclearinghouse.net/approvedgmo.shtml). However, progress has been stalled by ongoing legal battles challenging the use of modern biotechnologies. In Rwanda, field trials have been approved for late blight-resistant potato, cassava resistant to mosaic and brown streak viruses, and maize for insect resistance and drought tolerance (Genetic Literacy Project, 2024). Tanzania, on the other hand, amended its biosafety Regulations from a strict liability approach to a fault-based process, thus creating a more supportive environment for biotechnological innovations. The country has in the past allowed confined field studies of drought-tolerant and insect-resistant maize, as well as GM cassava for mosaic and brown-streak virus resistance (Lumpkin and Armstrong, 2009; ISAAA - AFRICENTER, 2016).

Whereas Uganda has made significant strides in GM crop research, the path to commercialization remains obstructed by regulatory and political challenges. The country lacks a robust legal framework, which has hindered the commercial cultivation of GM crops. The current biosafety regulatory framework is based on the Uganda National Council for Science and Technology (UNCST) Act of 1990. The law established the National Biosafety Committee (NBC) as a technical arm to handle applications for contained and confined research that require biosafety approval. The UNCST, through NBC, continues to approve and oversee all GMO-related R&D activities in the country (Ongu et al., 2023). Despite the lack of a legal framework to facilitate the commercialization of GMO products, the National Agricultural Research Organization (NARO) and other research institutions are actively engaged in GM research in Uganda. Confined field trials have focused on a variety of crops, including bananas that exhibit resistance to bacterial wilt and black sigatoka, cotton engineered for herbicide tolerance and insect resistance, maize with insect resistance and drought tolerance, and cassava resistant to Cassava Brown Streak Disease. Additionally, sweet potatoes are being studied for virus resistance, while potatoes are being developed for late blight resistance. Soybeans are also being researched for herbicide tolerance (Ongu et al., 2023; Zawedde et al., 2018). Furthermore, the country has initiated research on gene drive mosquitoes for malaria control, which is currently in the containment stage, with the technology being spearheaded by the Uganda Virus Research Institute in collaboration with the Target Malaria research consortium (https://targetmalaria.org/wp-content/uploads/2020/11/Uganda_FS_EN_National_Jul25.pdf).

Post-release monitoring of GMOs in the region, in relation to the responsible institution, monitoring period, and provisions in law, is summarized in Table 1. Frameworks for monitoring GMOs in the Eastern and Central African region are at various levels, with only Kenya having developed a specific post-release monitoring manual to guide the process (NBA, 2021). The Kenyan biosafety law and its subsidiary legislation provide for monitoring following the introduction of GMOs into the environment (Kenya, 2009; Biosafety Regulations, 2011). A permit issued under these Regulations is valid for an initial ten (10) years, and a renewal for a further 10 years is required. The renewal is pegged on successful monitoring, whose data forms the basis of extension, after which the GMO may continue to be released without further approval from the biosafety authority, assuming no adverse effects are reported. The post-release monitoring manual serves as a guide to applicants and other regulatory agencies on the requirements for environmental monitoring. The manual provides for CSM and GS based on the regulator’s pre-approved monitoring plan. Additionally, the manual describes data sources for GS, including farmers’ questionnaires, published scientific literature on related GMOs, data and information from other government agencies, and a farmers’ complaint system maintained by the permit holder. CSM, on the other hand, is hypothesis-driven and may involve a specific experiment designed to evaluate a specific risk. The applicant is supposed to submit a monitoring report on an annual basis. While the primary responsibility of post-release monitoring lies with the applicant, this does not stop the authority and other relevant agencies from carrying out their own independent monitoring to corroborate the applicant’s findings. Kenya rounds off its fifth year of Bt cotton commercialization, but no publicly available post-release monitoring report was available for further scrutiny by the time of publication. The draft National Biosafety Framework of 2004 in Tanzania has provided for monitoring, where the applicant is mandated to carry out monitoring and evaluation of risks continuously for a period commensurate with the life cycle of the species for any approval granted for import, transit, contained use, release, or placing on the market of a GMO. The framework has identified two types of monitoring schemes relevant to environmental releases. These include confirmatory monitoring required by the government for any assumptions made during risk assessment and voluntary monitoring, which can be undertaken by the applicant to provide further information for their own use. The competent authority in Tanzania, in partnership with other agencies, is tasked with the responsibility of coming up with a monitoring plan on a case-by-case basis, taking into account the environmental risk assessment and risk management, and the cost of monitoring for each release.

**TABLE 1:** Provisions for post-release monitoring in eastern and central Africa.

Country	Competent authority	Institutions/Parties with primary responsibility for monitoring	Type of monitoring	Monitoring period	Specific laws/Regulations or guidelines
Kenya	National biosafety authority (NBA)	Applicant	Case-specific, general surveillance, and compliance monitoring	Two consecutive periods of 10 years (20 years total) Monitoring reports submitted annually	Biosafety Act 2009; biosafety (environmental release) regulations (2011); manual for post-release monitoring, 2021
Uganda	National council for science and technology (UNCST)	Applicant	No specified approach	No defined timelines	National biotechnology and biosafety policy, 2008
Tanzania	Office of the vice president	Not specified	General surveillance, case-specific, compliance, and voluntary monitoring	No defined timelines	National biosafety framework for Tanzania- draft, 2004 Environmental management (biosafety) amendment regulations, 2015
Ethiopia	Environmental protection authority of the federal democratic republic of Ethiopia	Applicant	No specified approach	No defined timelines	Biosafety proclamation no. 655/2009, amended in 2015 Directive No. 08/2018) Draft biosafety framework, 2007
Rwanda	Rwanda environmental management authority (REMA) National biosafety committee	Applicant	No specified approach	Monitoring is for 20 years	Biosafety law (No 025/2024 OF 16/02/2024) Ministerial order No 001/MOE/25 of 13/01/2025 on the application for a permit for living modified organism-related activities
Cameroon	Minister of environment, nature protection, and sustainable development (MINEPDED)	Not specified	Not specified	Proportional to the organism’s life cycle	Biosafety law No 2003/006 of 21 April 2003 Biosecurity law No 2025/006 of 25 April 2025 Decree No. 2007/0737/PM of 31 May 2007

Ethiopia has a Biosafety Proclamation No. 655/2009, which was amended in 2015, giving rise to the Biosafety (Amendment) Proclamation No. 896/2015 (Federal Democratic Republic of Ethiopia, 2015). The country has developed several directives, notably a Directive to Determine the Content of an Application for Undertaking Deliberate Release of Modified Organisms (Federal Democratic Republic of Ethiopia, 2018), which is in place. The Directive No. 08/2018 (Part three, 12) spells out the responsibilities of the applicant in relation to monitoring. Key among them include the provision of methods for tracing GMOs and monitoring their effects, techniques for detecting the transfer of introduced genes to other organisms, and methods for detecting aberrant expressions of traits and their causes.

Rwanda has established a 20-year post-release monitoring requirement in its laws (Ministry of Education, 2026). Notably, after the lapse of the proposed monitoring period and satisfactory data, the GMO may continue to be introduced into the environment without further consideration. The competent Authority and the National Biosafety Committee are responsible for the overall monitoring, risk management, and post-release monitoring of GMOs. The applicant or the permit holder has the primary responsibility for post-release monitoring in Rwanda, but this does not rule out the involvement of other government agencies in carrying out their independent monitoring.

In Cameroon, the Biosecurity Law, 2023, establishes a broader framework for a national surveillance system and allows for the creation of biosecurity control areas for intensive monitoring in response to threats. Although the Cameroonian law and its subsidiary decree are ambiguous on the approaches to post-release monitoring, this is provided for through mechanisms tied to risk management, mandatory reporting, and regulatory oversight (Republic of Cameroon, 2003). The law does not provide for specific post-release monitoring timelines. In Uganda, while not directly focusing on GMOs, the National Environmental Act, 2019, obligates the applicant to undertake monitoring of projects in a regular and timely manner and with frequency commensurate with the nature of the project (Uganda, 2019). In the absence of a standalone Biosafety law, this requirement may inform any future development of post-release monitoring guidelines.

### 3.2 Provisions for post-release monitoring in west Africa

Several institutions in Nigeria are actively undertaking research on GM crops in collaboration with international organizations. Ongoing research includes the development of GM cassava with elevated levels of iron and zinc for enhanced nutritional and disease resistance (Narayanan et al., 2020). Research on soybeans for herbicide tolerance and late blight resistance in potatoes has continued over the years. The country has approved Bt cotton, Bt cowpea, and insect-resistant and drought-tolerant maize for commercial release in the years 2018, 2019, and 2024, respectively (Ongu et al., 2023). Research and development of GMOs in Ghana are being carried out by the Crop Research Institute (CRI) of the Council for Scientific and Industrial Research (CSIR), which has conducted trials on sweet potato for nutritional enhancement, rice for nitrogen and water use efficiency, and salt-tolerant (NEWEST) and Bollworm-resistant cotton (Bt cotton). On the other hand, the pod borer-resistant cowpea (Bt cowpea) was developed and released by the Savannah Agricultural Research Institute (SARI) of CSIR (Gakpo and Baffour-Awuah, 2024; AATF, 2025; USDA, 2017). Ghana approved the commercial release of Bt cowpea in 2022, with seed distribution to farmers for commercialization carried out in 2024. In May 2024, a significant legal case that opposed the introduction of GM products was rejected by a court in Ghana. This decision represented an important step forward for the commercialization of GM crops in the nation (ISAAA, 2024). In Burkina Faso, Bt cotton was approved for commercial use in 2008. However, in 2016, this approval was suspended due to numerous challenges (Dowd-Uribe and Schnurr, 2016). Although no commercial cultivation of Bt cotton is currently taking place in Burkina Faso, recent reports indicate that approval for the cultivation of Bt cotton hybrids has been granted for the period 2024–2033 (ISAAA, 2025b). Efforts are also being made to move Bt cowpeas from field trial to environmental release (Gakpo, 2020; Komen et al., 2020). Burkina Faso has also been involved in the Target Malaria Project, which has been involved in the development of Gene Drive Modified Mosquitoes (GDMMs) for malaria control in Africa. However, this project was halted in 2025. While there are no records on GM crop improvement in Mali, the country is working on the development of GM mosquitoes. In the case of Côte d’Ivoire, Benin, Togo, Senegal, and Niger, no approvals have been granted for GMO field trials, commercial releases, or imports.

Post-release monitoring of GMOs in the region with respect to the responsible institution, monitoring period, and provision in law is summarized in Table 2. Post-release monitoring frameworks for GMOs in the West African region are quite varied. In Nigeria, for instance, the Biosafety Act has mandated the competent authority to oversee monitoring of human health and the environment to determine the potential impacts of GMOs. Monitoring the spread and behavior of any released GMO according to the Biosafety Act is the responsibility of the applicant, where monitoring reports are required to be submitted on an annual basis to the Minister through the National Biodiversity Management Agency (Nigeria, 2015). The Biosafety Act specifies monitoring periods as being commensurate with the organism’s life cycle, where trees are to be monitored for 150 years, animals or microbes for 30 years, and other perennial plants with shorter life spans than trees to be monitored for 30–50 years. The National Biosafety Regulations also specify monitoring requirements for the environmental release permit. GMOs deemed to pose no risk to human or animal health, or the environment, after 20 years of monitoring may be released without a new permit, as long as the agency is notified every 5 years about their continued release. During the monitoring period, permit renewals must be sought, and these are granted for 10 years. Subject to compliance with these Regulations, the agency may renew the permit for such a shorter period as it may deem fit, stating in writing the reasons for the decision (Nigeria, 2017).

**TABLE 2:** Provisions for post-release monitoring in west Africa.

Country	Competent authority	Institutions/Parties with primary responsibility for monitoring	Type of monitoring	Monitoring period	Specific laws/Regulations or guidelines
Ghana	National biosafety authority	National biosafety authority	General surveillance and farmers’ feedback systems	No defined timelines	Biosafety Act, 2011 Biosafety (management of biotechnology) regulations, 2019
Nigeria	National biosafety management agency (NBMA)	Applicant	Not specified	Equivalent to the life cycle of the relevant species, or as prescribed Periodic monitoring reports 150 years (trees), 30 years (animal or microbe) or other perennial plants with shorter life spans than trees (30–50 years)	NBMA Act, 2015 (amended in 2019) National biosafety regulations, 2017 National biosafety policy, 2018
Burkina Faso	National biosafety (agency agence nationale de biosécurité, ANB)	National biosafety authority	Not specified	Commercial releases are monitored for 10 years	Biosafety law, 2012
Mali	National biosafety agency (agence nationale de biosécurité, ANB) Ministry of the environment	Not specified	To be determined on case by case basis	Dependent on the life cycle of the species Long-term monitoring is to be carried out for 30 years	Biosafety law 08–042 of 2008 Decree No. 10–683/P-RM (on the national biosafety committee) Decree No. 10–682/P-RM (on GMO experimentation)
Senegal	National biosafety authority and national biosafety committee (2009); scientific committee (2017)	Not specified	Not specified	No defined timelines	Biosafety Act 2009–27 (amended in 2022)
Ivory coast (cote d’ivoire)	National biosafety committee (comité national de la biosécurité, CNB)	Not specified	Not specified	No timelines provided	Law No 2016–553 of biosafety, 2016
Benin	Ministère de l environnement de la Salubrité et du développement durable	Not specified	Not specified	No defined timelines	Biosafety law 2021–01
Togo	Ministry of environment and forest resources	Not specified	Not specified	No defined timelines	Biosafety law, 2009 (law N 2009–001 009) Regulation No 0029/MEDDPN/SG/DRF of 24 March 2020
Niger	Ministère de l environnement de la Salubrité et du développement durable National technical and scientific biosafety committee (CTSNB)	Not specified	To be specified in future decrees	No defined timeline	National biosafety law (2019–48) ECOWAS regulation C/REG…/…/ 13… relating to biosafety in west africa UEMOA regulation No 007/2007/CM/UEMOA.

The Biosafety Act in Ghana provides for the monitoring of GMOs introduced into the environment where there is uncertainty regarding the level of risk (Ghana, 2011). The Biosafety Guidelines for handling requests for use of GMOs state that it is the responsibility of the applicant to continually collect information, conduct monitoring, and report as specified in the terms and conditions (National Biosafety Authority Ghana, 2016). The country has no defined timelines for post-release monitoring, but for commercial releases, general surveillance and farmer feedback systems are used to track long-term performance and environmental effects. Environmental release approvals last 10 years and are renewable. Applicants may seek GMO deregulation by providing evidence of minimal risks to humans, animals, and the environment. The Regulations assign overall monitoring, risk management, and environmental release responsibilities for GMOs to the Authority (Ghana, 2019).

Burkina Faso’s experience with transgenic cotton, specifically Bt cotton, offers valuable insight into the complexities of adopting agricultural biotechnology in Africa. The National Biosafety Agency (ANB) is responsible for implementing the Biosafety Law, including the approval, monitoring, and enforcement of GMO-related activities. The agency has also established a National Biosafety Committee, an advisory body that assists the ANB in decision-making processes regarding GMO applications and monitoring. The ANB is tasked with ongoing surveillance of GMOs post environmental release, and this includes monitoring for unintended effects on the environment and human health. Regular Environmental Impact Assessments (EIAs) are supposed to be conducted to assess the long-term impacts of GMOs on ecosystems and biodiversity. The duration of monitoring is directly linked to the scope of the authorization, with full environmental and commercial releases mandating long-term monitoring plans, like the 10 years specified for the authorization of Bt cotton, to assess cumulative and long-range effects.

The monitoring period for GMOs in Mali following full environmental release is defined by the life cycle of the species, determined on a case-by-case basis. Notably, the law mandates a specific long-term commitment for released GM animals, requiring a monitoring period of at least 30 years. The biosafety law in Côte d’Ivoire outlines the procedures for obtaining permits for any activity involving GMOs; however, implementing texts have yet to be put in place. Although countries such as Benin, Togo, Senegal, and Niger have biosafety laws in place, these countries have yet to approve any GMO field trials, commercial releases, or imports. Their respective laws have provided for requirements such as mandatory post-release monitoring plans, reporting obligations, and the protection goals broadly stated as biodiversity, human, and animal protection. The timelines and the monitoring approach have not been specified.

### 3.3 Provisions for post-release monitoring in southern Africa

South Africa remains the pioneer in GM crop adoption on the continent with the commercialization of GM cotton, maize, and soybean (ISAAA, 2018; Turnbull et al., 2021). In 1997, South Africa became the first country in Africa to approve commercial production of insect-resistant cotton (Gouse, 2009). This was followed by the approval of Bt maize and herbicide-tolerant soybean in 1998 and 2001, respectively. Cotton and maize varieties with stacked events (insect resistance and herbicide tolerance were subsequently released beginning in 2006. Since the first commercialization of GM crop, a total of 27 general release permits have been granted for commercial planting in South Africa (SANBI Report, 2021). These encompass traits relating to insect resistance, herbicide tolerance, and, most recently, the drought tolerance trait. Approvals have also been granted for the commercial release of three GM animal vaccines (5 permits), specifically for poultry diseases. Currently, South Africa has an estimated 2.74 million hectares under GM crop cultivation annually. In the last 20 years, permits for CFTs have been issued for wheat, potato, sugarcane, strawberry, canola, tomato, apple, groundnut, cassava, and sweet potato (SANBI Report, 2021). In Eswatini, Confined field trials for Bt-cotton were approved in 2016, with their subsequent commercialization in 2018. In Malawi, Bt cotton was approved for commercialization in 2018 following the successful completion of confined field Trials. In Mozambique, confined field trials with maize to evaluate drought and insect resistance have been ongoing since 2017. In Zimbabwe, Bt cotton trials have been going on since, but as of 2025, no research trials were openly available, and commercial release is yet to take place. There was no publicly available information on the development of GM crops in Zambia or Namibia.

Post-release monitoring of GMOs in the region, in relation to the responsible institution, monitoring period, and provisions in law, is summarized in Table 3. Post-release monitoring frameworks for GMOs in the Southern African region are at various levels, with only South Africa having a detailed post-release monitoring framework. In South Africa, monitoring under the GMO Act is complemented by environmental monitoring that is coordinated by SANBI, as well as food safety surveillance by the Food Control Section of the Department of Health. The Department of Environmental Affairs (DEA), which implements the National Environmental Management Biodiversity Act, 2004, confers on SANBI the responsibility to monitor and report on the environmental impacts of GMOs released into the environment. Post-release permit specified monitoring conditions are also undertaken by the applicant on an annual basis. To ensure industry compliance with the Regulations and promote good practices and stewardship, the South African National Seed Organization (SANSOR) also carries out independent monitoring (SANBI Report, 2021). The South African GMO Act includes provisions for post-market monitoring, which is done largely for compliance purposes and is carried out by authorized inspectors. Permits issued also set conditions for post-market monitoring to be performed by permit holders who are required to submit annual reports.

**TABLE 3:** Provisions for post-release monitoring in southern Africa.

Country	Competent authority	Institutions/Parties with primary responsibility for monitoring	Type of monitoring	Monitoring period	Specific laws/Regulations or guidelines
South Africa	Department of agriculture, forestry and fisheries (DAFF) Department of environmental affairs (DEA) South african national biodiversity institute (SANBI) South african national seed organization (SANSOR)	SANBI	General surveillance, case-specific, and compliance monitoring	Annual monitoring	GMO Act, 1997 GMO regulations, 2010 National environmental management biodiversity Act, 2004
Zambia	National biosafety authority	Applicant or their successor	The scope and frequency are to be defined in the permit	At least 30 years for plants, animals, and microorganisms At least 150 years for trees Other perennials with a shorter life span compared to trees is 30–50 years	Biosafety Act, 2007 National biotechnology and biosafety policy, 2007
Malawi	Environmental affairs department National biosafety regulatory committee (NBRC)	Applicant	The scope and monitoring parameters are to be outlined in the permit	Frequency and duration to be specified in the approval permit	Biosafety Act, 2002 Biosafety (management of genetically modified organisms) regulations 2007
Namibia	National commission on research, science and technology (NCRST) Biosafety council	Applicant	General surveillance Case specific monitoring	To be specified in the approval permit	Biosafety Act, 2006
Mozambique	National biosafety authority (ANB) Interinstitutional biosafety group (GIIBS)	Applicant	To be specified in the approval permit	To be specified in the approval permit	Decree 6/2007 (later replaced by decree 71/2014)
Zimbabwe	National biotechnology authority (NBA)	Not specified	Not specified	Not specified	National biotechnology authority Act of 2006, national biotechnology policy, 2005
Eswatini	Eswatini environment authority (SEA National biosafety advisory committee (NBAC) Biosafety registrar	Applicant	To be specified in the permit	To be specified in the permit	Biosafety Act of 2012, amended in 2020 Biosafety policy, 2006 Draft regulations 2023

The Biosafety Act of 2012 serves as the foundation for the regulation of GMOs in Eswatini. The law outlines the requirements for the importation, transit, handling, and use of GMOs to protect human health and the environment. The Act provides that in instances where there is uncertainty regarding the level of risk, this may be addressed by requesting further information on the specific issues of concern or by implementing appropriate risk management strategies and/or monitoring the GMO in the receiving environment. The applicant is solely responsible for monitoring the impacts of GMOs in their receiving environments.

In Namibia, the National Commission on Research, Science and Technology (NCRST) is mandated to administer the biosafety law and serves as the secretariat to the Biosafety Council. One of the specific functions of the Council is to undertake or promote research in connection with risk assessment and the biosafety of GMOs. The Namibian law proposes that any dealings that require authorization by a permit and involve the intentional release of a GMO or GMO product into the environment requires that the applicant must specify the proposed strategies and procedures to be used in monitoring any anticipated or potential effects of a such release, or an explanation of the reason why monitoring is not required (Namibia, 2006). The Guidelines for Placing Genetically Modified Food or Feed on the Market under the Biosafety Act state that environmental release permit holders are required to submit monitoring reports at such intervals as set out in the permit or as determined by the Council based on the results of the environmental release. The report to the Council on the results of the environmental release must include at least a post-release evaluation of the risks to the health and safety of humans and animals and the environment. Monitoring takes effect after the introduction of a GMO into the environment.

The Biosafety Act of 2002 is the primary legal instrument governing the regulation of GMOs in Malawi (Malawi, 2002). The law outlines the requirements for handling, transporting, using, and monitoring of GMOs. The National Biosafety Regulatory Committee (NBRC) is responsible for overseeing the implementation of the Act and ensuring compliance with biosafety regulations. The Biosafety law states that the applicant should provide for methods for tracing GMOs and for monitoring effects and provide for the duration and frequency of monitoring. Further reference to monitoring is with respect to when there is uncertainty in level of risk during risk assessment at which time it is expected that further information may be sought from the applicant or be addressed by implementing appropriate risk management strategies and/or monitoring.

The competent authority in Mozambique is supported by the Inter-Institutional Biosafety Working Group (GIIBS), which receives applications and forwards them to the competent bodies from the relevant sectors. The relevant sectoral entities provide technical advice and monitor and supervise the implementation of approved activities and are drawn from the Ministries of Agriculture and Rural Development (MADER), Health (MISAU), Land and Environment (MITA), and Inland Waters and Fisheries (MAIP). The Authority is also supported by Technical and Scientific Committees, which conduct technical assessments of the applications, risk assessment, and submit the reports to the GIIBS secretariat. Biosafety law requires a monitoring plan during the application process, which may include both GS and CSM. The applicant should ensure the implementation of monitoring and also facilitate the designated inspectors to carry out enforcement activities. The law in Zambia has provided for monitoring where the spread and behavior of any released plant or micro-organism, GMO shall continue for at least 150 years in the case of trees, and for at least 30 years in the case of annuals and microorganisms. The monitoring duration for perennials, with shorter life spans than trees lie somewhere in between. The user who is responsible for releasing the GMOs or their successor shall provide annual reports to the Authority. Any released animal that has been genetically modified will be monitored for at least 30 years (Zambia, 2007).

### 3.4 Provisions for post-release monitoring in northern Africa

In this region, Sudan has the most advanced system for regulating GMOs in the region, with the biosafety and legal frameworks in place to oversee the entire process of handling GMOs, including post-release monitoring. The country has experience with commercial GMO cultivation, notably with the approval and widespread adoption of Bt cotton since 2012. GMO research is conducted under the supervision of the National Biosafety Committee. The Biosafety Law in Sudan establishes a National Biosafety Council or Board, which forms a Biosafety Technical Committee responsible for all technical aspects of biosafety. This includes permit duration restrictions, reporting mechanisms, remedial measures, monitoring procedures, and other risk management strategies.

In Tunisia, the country has a National Strategy and Action Plan on biosafety; however, the National Biosafety Law is yet to be adopted. The National Biosafety Committee was established to oversee decisions on biosafety matters. No published ongoing GMO research and development or environmental release approval is currently in the country. In Egypt, a GM crop (Bt maize) was approved for environmental release in 2008. However, in 2012, open cultivation of the GM crop was suspended (Gakpo, 2019). With regards to the biosafety regulatory framework, the country has a draft law since 2006, which is yet to be approved by the cabinet.

Post-release monitoring of GMOs in the region with respect to the responsible institution, monitoring period, and provision in law is summarized in Table 4. In Sudan, the Council has the authority to require that any GMO or its derivatives undergo an observation period aligned with its life cycle or reproductive period, at the applicant’s expense, before and after it is made available for use. Furthermore, the applicant must submit periodic reports on monitoring and assessing risks that arise after approved environmental releases are undertaken. Monitoring the spread and behavior of any GMO is mandated to last for at least 150 years for trees, and at least 30 years for annuals and animals. The person responsible for the release is required to prepare annual reports for the Board. There are no post-release monitoring provisions in Tunisia and Egypt.

**TABLE 4:** Provisions for post-release monitoring in northern Africa.

Country	Competent authority	Institutions/Parties with primary responsibility for monitoring	Type of monitoring	Monitoring period	Specific laws/Regulations or guidelines
Tunisia	Ministry of environment National agency of sanitary and environmental control products National biosafety committee	Not specified	Not specified	Not specified	No biosafety law, but there is a national strategy and action plan on biosafety
Egypt	Nature conservation sector of the egyptian environmental affairs agency	Not specified	Not specified	Not specified	Draft biosafety law for the release of genetically modified products, 2006
Sudan	Higher council for environment and natural resources National biosafety council	Applicant	Surveillance, monitoring, inspection	150 years for trees, 3 years for animals and annuals that live less than trees	National biosafety law 2010 Draft national biosafety framework, 2006

### 3.5 Experience with post-release monitoring in selected countries

To get further insights from countries that are already cultivating GMOs, an online survey was conducted targeting Kenya, Ethiopia, South Africa, Malawi, Eswatini, Nigeria, and Sudan. The survey, which targeted regulators from the countries cultivating GMOs, sought to understand whether post-release monitoring was a requirement in the approval process and its objective in the decision-making process. Applicants whose products are being cultivated in these countries were also recruited for the study. Feedback was received from seven of the eight countries surveyed. No responses were received from Sudan. From the survey it was established that in all the responsive countries, post-release monitoring was one of the approval conditions with the applicant expected to submit approved post-release monitoring plan prior to the commercialization of the GMO. In most of the countries surveyed, the applicant has the primary responsibility of monitoring GMOs once introduced in the environment, but the biosafety agency together with other relevant agencies (e.g., Kenya Plant Health Inspectorate Service) carries out independent monitoring to corroborate the findings of the applicant. However, in Ethiopia the regulatory agency has the primary responsibility for post release monitoring. South Africa has a unique situation in which in addition to the applicant having the primary responsibly and reports to the regulatory agency, SANBI has specific responsibility of monitoring impact of GMOs on biodiversity and reports directly to the Minister concerned. The post-release monitoring strategies included GS and CSM. The commonly used approaches for GS include farmers questionnaires, focused group discussion, key informants and farmers complaint system developed by the applicant. Case specific monitoring generally centered around monitoring resistance development in the target pests. Most countries require regular submission of post-release monitoring reports to regulatory agencies, which then share them with relevant authorities. These reports were not shared publicly or hosted on the websites of any of the countries or the biosafety clearing house. All countries use the reports to inform reviews of approval conditions and permit extensions.

## 4 Discussion

The importance of post-release monitoring of GMOs is well articulated by the CBD, which mandates Parties to monitor the components of biological diversity important for its conservation and sustainable use, and identify activities that are likely to have significant adverse impacts. The Cartagena protocol and the CBD guidance document (CPB, SCBD, 2000; CBD, 2016) also provide that in instances where there is uncertainty regarding the level of risk, this may be addressed by requesting further information on the specific issues of concern or by implementing appropriate risk management strategies and/or monitoring the LMOs in the receiving environment. This is of particular significance in the African biosafety and GMO oversight context because most African countries are signatories to the Cartagena Protocol. Treaty obligations are reflected in national biosafety laws, protection goals, guidance, and policies, including any related to the monitoring of GMOs in the environment.

The primary objective of post-release monitoring is to facilitate early detection and implement appropriate mitigation measures for any adverse effects of GMOs on humans, animals, or the environment. Through monitoring, the performance of an approved GM product can also be established (Andrade et al., 2014). Most African countries have developed responsive regulatory frameworks for GMOs, but the implementation of a comprehensive post-market monitoring framework is still evolving (McLean et al., 2003). Out of the 26 countries surveyed, 24 have provided for PRM in their laws, but only 2 give further guidance on how PRM should be undertaken. However, it’s worth noting that countries are grappling with a lack of a clear national monitoring plan that is reasonable within resource constraints, and have taken a highly precautionary approach (Andrade et al., 2014; SANBI Report, 2021; Pinheiro et al., 2023). Additionally, there is poor coordination among the various institutions involved in the review and monitoring of GMOs, and often, there is no free flow of information (SANBI Report, 2021; Rabuma et al., 2024). This has hampered the enforcement efforts due to the fragmentation of legislation applied in regulating GMOs. In this survey, which focused on the selected African countries, most indicated the need for post-release monitoring of GMOs, but additional guidance on how this should be carried out was only available in Kenya and South Africa. While South Africa designates a specific institution, SANBI, to carry out this role in addition to the Ministry of Agriculture, no further information is provided on whether and how or not such reports have been regularly submitted and used during the decision-making process. In their latest review, they attempt to advocate for a structured approach to post-release monitoring. This is the scenario in South Africa, which is the leading African nation in the adoption of GMOs (SANBI Report, 2021).

As part of the approval process for environmental release of GMOs in Kenya, an applicant is obligated to submit monitoring and stewardship plans before the permit is issued. In the case of commercialization of Bt cotton, the plan is to monitor the efficacy of the particular GMO through a field-based approach involving scouting sentinel sites and monitoring in commercial fields. The plan also proposed the use of the farmers’ complaint system as a complementary monitoring tool, allowing farmers to report complaints on the performance of the transgenic cotton. To establish the adequacy of the insect resistance management strategy for Bt cotton, the permit holder proposes resistance evolution and refuge compliance monitoring. Resistance monitoring is designed to detect early warning signs indicating potential increases in Cry1Ac and Cry2Ab2 (the modified traits introduced in Bt cotton) tolerance in field populations of African bollworms (Messéan et al., 2024; Hu et al., 2025). Additionally, the regulator has also developed a post-release monitoring manual to guide applicants, and it provides for GS, CSM, and compliance monitoring (NBA, 2021). The manual distinguishes between GS and CSM, with the latter meant to confirm the assumptions made in the ERA with regard to the possibility of insect resistance developing. No post-release monitoring report has been published since the adoption of Bt cotton in 2020.

To place the status of post-release monitoring of GMOs in Africa in the context of the practice in other countries, the experiences in the European Union and Brazil were reviewed as well. In the EU, post-market environmental monitoring of GMOs became mandatory through a resolution in 2001 (EC, 2001). According to the published guidelines, post-market monitoring was classified as either CSM or GS (EFSA, 2011; EFSA, 2006). Cultivation of the insect-resistant maize (MON 810) in the EU was approved under Directive 90/220/EEC by the Commission Decision 98/294/EC, and the event has since been introgressed into local varieties widely adopted in Spain and Portugal (Messéan et al., 2023). Most documented post-release monitoring experiences in the EU is related to the cultivation of maize, MON810, which was approved in 2003, with the post-release monitoring initiated from 2005 (Messéan et al., 2024). The consent holder reports the results of the insect resistance management, which focuses on monitoring resistance evolution and refuge compliance as part of CSM, as well as the results of GS to the European Commission and the EU Member States. EFSA has evaluated the annual post-market environmental monitoring reports on maize MON 810 corresponding to the 2009–2022 period (Messéan et al., 2024). The most recent review concluded that the reports did not point to new hazards or new scientific uncertainties that would change the conclusions of prior risk assessment and risk management recommendations for MON810. However, several recommendations were made to improve the post-release monitoring strategy. Statistical analysis on pooled results of 10 years (2006–2015) of post-market environmental monitoring on MON810 carried out based on farmer questionnaires, did not reveal any unexpected adverse effects associated with its cultivation (Bertho et al., 2020). In conclusion, the authors suggest that GS should be focused on literature searches and the farmer complaint system based on the extensive safety data package for MON 810, the robust weight of evidence demonstrating both its safety and benefits, and the history of safe use of MON 810 for 15 years in the EU. However, this suggestion has been criticized by EFSA on the basis that the system has not been fully developed (Messéan et al., 2024).

In contrast to the African member countries under review, the EU provides for an obligatory framework for post-release monitoring of GMOs. The primary responsibility is to the applicant who submits annual reports to EFSA for review. The applicant is expected to provide for case-specific and general surveillance. Post-release monitoring for MON810, which is the only crop approved for cultivation in the EU, has been carried out annually since 2009. It is unclear as to how much longer the reports are expected. It is also worth noting that the review of the post-release monitoring is publicized by EFSA and is accessible to all. While Kenya provisions for post-release monitoring of GMOs, which compare well with the EU guidelines, post-release monitoring reports have been submitted annually since 2021 (personal communication), but how such reports are going to be processed and shared with the public remains unclear. The biosafety regulatory system in Kenya prescribes public participation as a mandatory process in the decision-making process. It is therefore expected that such reports that inform decision-making be made public as well. This would also build trust between the public and the agencies tasked with the mandate of GMOs oversight. In addition, public reporting would go a long way in reducing possible litigations that have continued to surround Kenya’s GMOs debate where inadequate public participation has been cited (Law Society of Kenya v Attorney General et al., 2023).

In Brazil, CTNBio Normative Resolution No. 5 of 2008 stipulates that an application for commercial approval of a GMO in Brazil must include a post-commercial release monitoring plan. The primary objective of this plan is to monitor the potential impacts of extensive planting of the GM event and its derivatives, both on the environment and human and animal health. The post-release monitoring reports are supposed to be submitted to the regulatory agency on an annual basis for review for a period of 5 years (Pinheiro et al., 2023). In 2011, Normative Resolution No. 9 introduced alternative approaches for the post-release monitoring plan involving GS and a CSM scheme. GS includes reviewing pertinent technical documents to assess technology utilization, through accessible and appropriate communication channels, administering user questionnaires, as well as scrutiny of scientific articles published in peer-reviewed journals or in reports by governmental agencies and official notification systems for unexpected GM-related biosafety concerns. Any observed effects during the general surveillance involve filing a technical report, which is subsequently analyzed by CTNBio. If the agency deems the effect potentially adverse and causally linked to GMO usage, Normative Resolution No. 9 mandates the implementation of a CSM plan to run concurrently with the GS. Should the scientific study conducted under the CSM plan reveal that the adverse effect indeed poses a biosafety risk associated with the GMO, CTNBio is then tasked with making a critical decision, which may involve either suspending the commercialization of the GMO or canceling the prior approval granted by the agency.

However, in a subsequent development in 2021, CTNBio Normative Resolution No. 32 exempted GM events that exhibit negligible effects during the risk assessment stage from presenting a post-marketing monitoring plan. In a review of 5-year post-release monitoring reports for GM eucalyptus in Brazil, GM eucalyptus event H421 (for increased biomass), no adverse effects associated with biosafety risks were observed. Given the absence of adverse effects noted during the surveillance monitoring, event H421 was not required to undergo a CSM (Pinheiro et al., 2023). According to the Brazilian Biosafety law, the applicant is responsible for the post-release monitoring of GMOs. Monitoring is also a responsibility of some federal agencies involved in registration and inspections from the Ministries of Health, Agriculture, and the Environment.

Comparison between the EU and Brazilian provisions for post-release monitoring shows that while both frameworks provide for case-specific and general surveillance, in the Brazilian system, the initial monitoring is for GS, which may result in CSM should an adverse effect be realized. The primary responsibility is on the applicant who submits annual reports to CTNBio for review for a period of 5 years. The Brazilian system provides for review of post-release monitoring conditions depending on the annual reports, with the possibility of subsequent exemption from post-release monitoring. The possibility of exemption from post-release monitoring sets the Brazilian system apart from the EU and the African countries, where there is no such exemption.

Due to the current inadequacies in biosafety regulatory and monitoring frameworks surrounding GMOs, many African nations exhibit a deficiency in regulatory measures governing the utilization of these modern biotechnologies when compared to other regions globally (Sadikiel Mmbando, 2024). There is little consensus among the sampled countries regarding the recommended management or monitoring requirements for GM crops once they are introduced into the environment. This situation is partly attributed to the lack of well-defined environmental protection objectives in many of these nations. Furthermore, even where regulatory frameworks are in place, they often prove impractical due to limited human and financial resources (Bartsch et al., 2006; Sanvido et al., 2012; Dolezel et al., 2024). Additionally, the monitoring timelines outlined in most policies are unrealistic, as they provide no distinction for different types of GMOs. For example, monitoring GMOs for 20 years before deregulation is certainly not feasible, practical, or meaningful for those with very short reproduction cycles, such as GM mosquitoes, yeast, or bacteria (Andrade et al., 2014).

A thorough analysis of different frameworks for managing GM crops after their commercialization shows a lack of flexibility and adaptability to rapid technological changes. Such frameworks have not identified key actors and defined their specific roles with respect to the follow-up of GMOs once introduced into the environment. Conducting risk assessments before approving environmental releases is essential before setting up monitoring requirements, as this helps reduce duplication, cut monitoring costs, and optimize resource use in a transparent and defensible way. The selection of GS tools, such as farmers’ questionnaires, should be responsive and act as an early warning system since farmers interact with GMOs throughout their cropping cycles (Messéan et al., 2023). Additionally, a centralized repository for reports on GMO risk assessment, management, and monitoring data could help inform and guide compliance levels and future approval decisions.

## 5 Conclusion and policy recommendations

Despite decades of GM crop cultivation, concerns persist in certain sections of society regarding their environmental impact (Gbashi et al., 2021). Monitoring concepts and guidelines have not been implemented in practice in most countries, and where commercial cultivation of GM crops, particularly those with Bt or insect resistance traits, is ongoing, the focus has been on identifying the development of insecticide resistance in the target pests in growing and surrounding areas. Other potential adverse effects have largely not been addressed, thus calling for the need to re-examine the existing monitoring frameworks to determine their broader applicability for detecting other potential adverse effects on the environment (Dolezel et al., 2024). This review reveals regulatory gaps, particularly the absence of case-by-case risk assessment and identification of specific monitoring indicators, parameters, and methodology, as well as unclear monitoring responsibilities, adaptive monitoring, and potential for deregulation. While the study was focused on selected African countries, it highlights the importance of product and use case-specific monitoring strategies aligned with national laws and protection objectives to ensure the safe and responsible use of GMOs.

Post-release monitoring is a key component of GMO regulation that ensures continued oversight following an approval for environmental release to ensure ongoing product safety and efficacy (Mbabazi and Maredia, 2019). Monitoring frameworks vary from country to country, but regardless of the approach, they are all geared towards human, animal, and environmental protection. The need to monitor should be provided for in the national biosafety legislation, outlining clear objectives, scope, and a duration that is adequate for any unanticipated adverse effects to be detected. However, it is important to note that post-release monitoring should ideally be on a case-by-case basis, reflecting available best practices and principles laid down by the Cartagena Protocol on Biosafety.

Post-release monitoring and stewardship plans are a prerequisite to most approvals for commercial cultivation of GMOs. These plans should clearly define the assessment endpoints and corresponding measurement endpoints (Sanvido et al., 2012; Dolezel et al., 2024). Whereas CSM and GS are widely recognized approaches to post-market monitoring, a case-by-case approach that is responsive enough to detect causally linked adverse outcomes reliably and quickly can be adopted. A distinction between what CSM and GS are meant to achieve, with clear justification as to why one should be undertaken, needs to be at the heart of every GMO monitoring program. While general monitoring principles and approaches remain applicable, specific monitoring designs, indicators, parameters, and methods must be aligned with the GMO under consideration, taking into account the specific exposure routes and potentially affected habitats and species.

Countries that have adopted GMOs might consider committing adequate resources towards comprehensive monitoring and enforcement activities. Investing in developing technical expertise nationally and regionally for effective risk assessment and monitoring, and deliberate efforts to engage and educate the public for building trust and support for GMO regulations can be explored. Effective utilization of the limited resources may also be realized through a coordinated approach to post-release monitoring. Thus, post-release monitoring of GMOs need not fall on a single actor, as seen in many countries. Instead, it can aim to improve the participation of all relevant stakeholders through a monitoring network that coordinates the monitoring approach. This avoids fragmentation and promotes credibility and consistency in reporting as well as efficient utilization of resources. While the primary responsibility of post-release monitoring remains with the applicant, the involvement of other stakeholders is well structured, with the National Competent Authority retaining the coordinating role. The Competent Authority, while reviewing the reports, may engage relevant expertise so as to decide on any mitigation measures.

Exemptions from monitoring, as well as the exemption from risk assessment in some cases, may be considered. Countries like Brazil have included exemptions in their system (Andrade et al., 2014). However, this consideration can be case-specific, taking into account the nature of GMOs being released into the environment. Additionally, the monitoring period as well as the parameters to be monitored may be reviewed by the regulator depending on the reports accumulated over time. Additionally, data generated through years of intense monitoring of GMOs can inform plans to monitor related events, especially where the receiving environments are comparable. This approach is similar to what has globally become accepted as data transportability in risk assessment of GMOs to reduce the time and cost of generating additional data with no value to the decision-making process (Chege et al., 2024; Mbabazi and Maredia, 2019).

## 6 Limitations of the study

The implementation status of the post-release monitoring provisions could not be fully verified in many countries, as internal monitoring reports were either unavailable or unpublished. The review relied on publicly accessible documents, which may have missed more recent updates or unpublished guidance. The language barrier restricted the inclusion of documents not available in English and those that could not be easily translated.
